# Asian/Pacific Island Technology and Health

**DOI:** 10.31372/20180304.1001

**Published:** 2018

**Authors:** Reimund Serafica

Nurses are masters at multitasking—that is performing several nursing interventions simultaneously during a patient encounter. While the new technologies such as smart pumps, bar-code medication, administration systems, electronic health records (EHRs), wearables, and smartphones being introduced into our practice environments are designed to increases efficiency, promote safety, and streamline the work of nursing, we also need to see what is current and emerging in the research arena. We need to continue to seek new evidence-based practice to care effectively for the Asian/Pacific Islander populations and promote a healing environment while incorporating the advantages and efficiencies that technologies provide.

Information and communication technologies have become an integral part of our lives. Many of us, spend much of our time with technological devices for professional and for personal uses. Collaborative research practice encourages researchers from different health-related professions to work together on creating and disseminating new knowledge to support evidence-based practice (Yen, 2018). Such collaborations allow new knowledge or interventions to be more interpretable and applicable to real-world settings. One of the National Institute of Nursing Research’s (NINR) promoting innovation plans is the use of technology to improve health, capitalizing on various technologies to deliver affordable health promoting interventions across populations and settings. Innovative technologies play an imperative role in advancing health care and nursing science that generate unique and culturally sensitive interventions to deliver tailored care and real time health information to patients, families, clinicians, and communities (Grady & Gough, 2015). NINR continues to support research programs that are developing and refining technologies to improve risk assessment and identify potential interventions including rapid advances in data science, genomic and molecular research, as well as devices and software to improve health across the health sciences.

The use of mHealth, social media, various mobile technologies, smart phones, and smart homes are only few of the things that are already in existence and continue to serve and impact the health status of various populations. Technologies that provide support for the collection and analysis of clinical data are also evolving. As new technologies are developed, we will experience more rapid knowledge dissemination than was previously possible with more traditional forms of research, especially the randomized clinical trial. Research samples will no longer be limited by the researcher’s access to subjects. The use of cloud-based storage for instance, has assisted several longitudinal studies recently and it is becoming increasingly popular (Udtha, Nomie, Yu, & Sanner, 2015).

This special issue presents seven distinct articles that intertwine with one another, from various scholars in the field, with a common goal to enhance the health of Asians and Pacific Islanders. The different interventions using various technology platforms, technology and health reviews, and technological theory development themes addressed not only the specific health needs but also include the cultural needs of this population. It is our hope that these carefully selected articles will continue to motivate us to learn more and perhaps delve more, design more, and use more technology in our research, practice, and education.

The first article, *Interactive CO-learning for Research Engagement and Education (I-COREE) curriculum to build capacity between community partners and academic researchers*, describes how to achieve a collaboration between the academic nurse researchers and culturally diverse community partners at an Asian community-based health and social services center to co-create a culturally safe integrative education and training curriculum, the I-COREE innovative project. The purpose was to design, implement, and evaluate the I-COREE curriculum to respond to community partner’s needs to create a culturally safe place. The integrative education and training curriculum facilitated building an academic–community partnership capacity for research engagement that included developing trust and rapport and addressing uncertainties in health-assistive technologies.

The second article, *Exploring challenges in conducting e-mental health research among Asian American women*, argues that although e-mental health services and resources are now available and despite the rapid growth of these tools and resources in the healthcare market, most of them have not been empirically tested. The article specifies the relevance and applicability of e-mental health services to Asian American women and also highlights the two main barriers in conducting e-mental health intervention research: recruitment and adherence. The recommended framework proposed by the authors would assist nurse researchers to develop culturally-sensitive recruitment and retention strategies for e-mental health intervention programs.

Our third article, *Health-assistive smart homes for aging in place: Leading the way for integration of the Asian immigrant minority voice*, declares that since the voice of minority communities has been underrepresented in the development of smart home’s artificial intelligence (AI) algorithms, the authors continue to challenge the need for more meaningful collaborations with Asian community leaders and members. Helping older adults remain independent in the setting of their choice is a complex multifactor endeavor. The recommendation is to combine the western (independent) perspective with the eastern (interdependent) perspective to be more culturally sensitive and appropriate for the aging Asian Americans that can benefit from the comfort of the smart home technologies.

The use of the photovoice technology as a method to capture the perceptions of the participants is employed in our fourth article, *Facilitators and barriers to being physically active in a rural Hawai‘i community: A photovoice perspective.* Photovoice is a participatory action research strategy by which people create and discuss photographs as a means of catalyzing personal and community change. In this qualitative study, major themes that emerged as facilitators to physical activity were: availability of outdoor amenities, accessibility to outdoor amenities, work duties, and cultural activities. Major themes identified as barriers to physical activity were: perceived safety, availability of built amenities, accessibility to existing built amenities, social norms, and the weather. This study recruited 13 participants from rural towns located on the north and northeast shores of the island of O‘ahu in the state of Hawaii showcasing the social-ecological impact on behavior using personalized technology.

The fifth article, *The use of social media and mEMA technology in comparing compliance rate among users*, centers on the concept of compliance. The study reports the results of two studies’ approaches to dietary monitoring using social media (Facebook) and mobile-based Ecological Momentary Assessment (mEMA) technology, to include the lessons learned for improving participant compliance. The purpose of the first study was to examine the feasibility, acceptability, and efficacy of using Facebook as a platform to document and promote health interventions. The purpose of the second study was to investigate the feasibility of mEMA’s new photo-capturing feature to analyze the participant’s dietary intake. The major aim of this study was to report participant compliance in dietary self-management (i.e., recording food intake) by comparing the results of participants using a social media platform to those using mEMA. Choosing the proper research design to increase compliance in Asian populations is imperative not only for attrition purposes but also for sustainability of life interventions when the study is completed.

The current available measures of postural control are lab-based or supervised, which may hinder timely symptom assessment for those individuals with mild traumatic brain injuries (mTBIs), including Asian populations, who do not seek initial screening post-injury. In this proof-of-concept testing study, *Proof-of-concept testing of a real-time mHealth measure to estimate postural control during walking: A potential application for mild traumatic brain injuries*, which is our sixth article, the authors introduce us to a real-time mobile health (mHealth) system to measure postural control during walking, which can be used as the home-based monitoring approach. The potential of the proposed real-time mHealth system for home-based symptom assessment and management of mTBI, may mostly benefit Asian and other nonwhite racial minority groups who appear less likely to access post-acute rehabilitation.

Finally, our last but certainly not the least seventh article, *How can nurses drive technologies of healthcare in the Asia-Pacific*? explores the possibility that nursing could also drive technological advancement in nursing care and health sciences and nor merely as end-users of technologies. The article stresses the importance of in caring for patients in an increasingly technological world and that nurses recognize that technological proficiency is an advantageous attribute and not as a substitute for caring but rather an enhancement of caring. To facilitate this process, the authors explain that nurses must ensure that technologies are adapted to nursing practice specifically during the design and developmental stage of healthcare technologies.

I have been extraordinarily blessed to serve as the Guest Editor for the Health and Technology special issue of the *Asian/Pacific Island Nursing Journal* under the mentorship of journal Editor-in Chief, Dr. Jillian Inouye. Through the articles in this issue we once again show the ways to research and write about how technology influences our work as scientists, researchers, educators, practitioners, and above all, as nurses. I am deeply grateful and appreciative to these authors for their loyalty and their continued research and scholarship that serve the Asian/Pacific Islander populations. Also I want to acknowledge and extend my sincerest gratitude to all the reviewers and to our journal manager, Ms. Pamela Wilson, for this issue and for all their expertise and patience for this process so necessary to putting this issue together. As I wrote the *Introduction* for this issue, health and technology research would not be possible without the willingness of our research participants to participate in our studies; our research collaborators in other disciplines; and our internal and external agencies that funded our studies. I praise their courage, admire their generosity, and give many thanks for their faith in us.

## Declaration of Conflicting Interests

The author declares no potential conflicts of interest concerning the research, authorship, or publication of this article.
